# Death by Lightning

**Published:** 1852-01

**Authors:** 


DEATH BY LIGHTNING.

A boy was killed by lightning, at Jacksonville, Ills., in August last, under circumstances somewhat novel. He was in the act of passing from one house to another when struck, and was within fifty feet of the one he had left. It was in a part of the town thickly settled; there was a lightning rod on the house to which he was going, two hundred feet distant, but none on the one nearest him; there were no trees or high objects of any kind nearer than the houses. The only marks upon the boy were a few small spots on the forehead; his eye-brows and front hair were considerably singed. His hat (a common straw hat), had a hole three inches in diameter knocked through the front crown; most of the straw was carried away, and around the edges of the hole *all the straws were pointing upwards.*

Prof. Coffin, who oommunicates the foregoing facts in the last numbe of Silliman's Journal, concludes, from the appearances of the hat, that the boy was killed by a *return stroke*, the electricity passing from one cloud to the other (there being two which approached in opposite directions,) and taking the earth and the boy in its circuit.

It is remarkable that this accident should have occurred in the open air in the midst of a town, and in this particular is perhaps unique. The houses West and South of the fatal spot are well supplied with lightning rods, and Prof. Coffin conjectures that the electricity might have passed silently from the cloud to the earth by these and accumulated at the very point where the boy was when killed.

